# Research Advances in Gametogenesis and Embryogenesis Using Pluripotent Stem Cells

**DOI:** 10.3389/fcell.2021.801468

**Published:** 2022-01-20

**Authors:** Yuxin Luo, Yang Yu

**Affiliations:** ^1^ Beijing Key Laboratory of Reproductive Endocrinology and Assisted Reproductive Technology and Key Laboratory of Assisted Reproduction, Ministry of Education, Center of Reproductive Medicine, Department of Obstetrics and Gynecology, Peking University Third Hospital, Beijing, China; ^2^ Stem Cell Research Center, Peking University Third Hospital, Beijing, China

**Keywords:** organoids, PGCs, PGCLCs, embryoids, *in vitro* induced gametes

## Abstract

The previous studies of human gametogenesis and embryogenesis have left many unanswered questions, which hinders the understanding of the physiology of these two vital processes and the development of diagnosis and treatment strategies for related diseases. Although many results have been obtained from animal studies, particularly mouse research, the results cannot be fully applied to humans due to species differences in physiology and pathology. However, due to ethical and material limitations, the direct study of human gametes and embryos is very difficult. The emergence and rapid development of organoids allow the construction of organoid systems that simulate gametogenesis and embryogenesis *in vitro*, and many studies have successfully established organoid systems for some parts of or even the entire processes of gametogenesis and embryogenesis. These studies typically start with the establishment of mouse models and then modify these models to obtain human organoid models. These organoid models can be used to obtain a better understanding of the signaling pathways, molecular mechanisms, genetics, and epigenetic changes involved in gametogenesis and embryogenesis and could also be applied to clinical applications, such as drug screening. Here, we discuss the formation of primordial stem cell-like cells (PGCLCs), and in vitro-induced gametes and embryoids using pluripotent stem cells (PSCs). We also analyze their applications and limitations.

## Introduction

Human gametogenesis and embryogenesis are complex processes characterized by dynamic changes in the structure and surrounding environment. Due to the scarcity of materials and ethical issues, the molecular mechanisms involved in these processes have not been fully explored. The use of animal models is a universal method that has long been and is currently used in life science and biomedical research, and sufficient research resources are available. Therefore, the use of animal models to study human gametogenesis and embryo development has yielded many achievements. However, human gametogenesis and embryogenesis are human-specific processes, and genetic and physiological differences exist between humans and animals, which hinders the further exploration of human gametogenesis and embryo development. Reproductive problems are also unique to human beings. The development of reproductive medicine is tightly associated with the understanding of signaling pathways and regulatory mechanisms in gametogenesis. Therefore, the use of human-specific models for research is needed, but the use of human gametes and embryos for research has many limitations. In recent years, the emergence and rapid development of organoids have attracted much attention. Organoids are three-dimensional miniature models of organs generated by stem cells [(pluripotent or adult stem cells, PSCs or AdSCs)], and to some extent mimic the structure and function of their counterparts *in vivo*. Different cell types originate from different developmental stages and undergo different developmental pathways. Therefore, AdSCs are mainly used to for the construction of adult tissue organoids. PSCs are capable of infinite self-renewal and differentiation into all cell types with the exception of extraembryonic tissues and are mainly used for studying developmental events. Therefore, organoids constructed with PSCs have a great advantage in simulating the early organogenesis of tissues and organs, particularly gametogenesis and embryogenesis. Much research progress has been achieved in the generation of reproductive organoids and embryo organoids through the use of PSCs and AdSCs to mimic gametogenesis and embryogenesis.

In this review, we discuss the recent research advances regarding *in vitro* induced gametogenesis and embryoids in mice and humans using PSCs, and briefly discuss their implications and prospects.

## Overview of Mouse and Human Gametogenesis *In Vivo*

Primordial germ cells (PGCs) are the first germ cell line in the embryo and the precursors of oocytes and sperm. The main processes of germ cell development include PGC development, germ cell specialization, and gametogenesis. In most mammals, PGC precursors were committed early, probably to protect these cells from somatic cell lineage differentiation signals. Signaling molecules from surrounding somatic cells induce the fate of PGC precursors and play a key role in PGC specialization. PGCs generally emerge before or during gastrulation. Due to the small number of PGCs, ethical issues related to early embryo development and reproduction, and technical difficulties, limited studies have investigated human PGCs. However, using other model animals, such as mice, much has been learned about reproductive development.

In mice, the extraembryonic ectoderm (ExE) and visceral endoderm (VE) produce bone morphogenetic protein 4 (BMP4) and other molecules (Lawson et al., 1999; Shirazi et al., 2012). On approximately embryonic day (E) 6.25–6.5, BMP4 induces the most proximal posterior epiblast cells to become transcriptional repressor B-lymphocyte-induced maturation protein 1 (*Blimp1*) (also known as PR domain-containing 1, *Prdm1*)-positive PGC precursor cells (Ginsburg et al., 1990; Lawson et al., 1999; Saitou et al., 2002). At approximately E6.75-7.0, *Prdm1-*positive PGC precursors differentiate into about ∼40 *Prdm1*-positive, developmental pluripotency-associated 3 (*Dppa3*, also known as *Stella*) positive PGCs within the extraembryonic mesoderm (ExM) (Ohinata et al., 2005; Saitou et al., 2005). In addition to *Prdm1* and *Dppa3*, PGC markers also include interferon-induced transmembrane protein 1 (*Ifitm1*) and *Akp2* (also known as tissue nonspecific alkaline phosphatase) (Saitou et al., 2002). At E7.5, PGCs initiate migration, from the posterior streak to the endoderm, extraembryonic endoderm (ExEn), and allantois (Anderson et al., 2000). This initiation of mouse PGC migration was thought to be regulated by IFITM1 (Tanaka et al., 2005), but another study showed that the deletion of IFITM1 does not affect reproductive development (Youngren et al., 2005), thus, the role of *Ifiitm1* needs further investigation. At E8-9.5, PGCs migrate along the endoderm (future hindgut), and this migration is accompanied by global epigenetic reprogramming, which mainly includes demethylation of DNA and histone3/lysine9 dimethylation (H3K9me2) at around E8.0, and the upregulation of H3K27me3 at around E8.75 (Seki et al., 2005). At E9.5, PGCs migrate through the hindgut to the mesoderm, and this migration is followed by bilateral migration to the gonadal ridges to result in the formation of embryonic gonads at E10.5–11.5. *Dazl* and *Ddx4*, which are germline genes, start to show expression at E10.5. At E13.5, PGCs undergo sex differentiation and acquire parental epigenomes (Lin and Capel, 2015). In male mice, PGCs enter into mitotic arrest during the fetal period and differentiate into pro-spermatogonia (Hilscher et al., 1974), which result in the acquisition of an androgenic epigenome (Kanatsu-Shinohara and Shinohara, 2013; Kubo et al., 2015). After birth, pro-spermatogonia differentiate into spermatogonia. On postnatal day 10, most of the spermatogonia enter into spermatogenesis. At approximately 3 weeks after birth, the first population of haploid sperm cells is produced (Bellve et al., 1977), and a small group of spermatogonial stem cells (SSCs) maintain spermatogenesis during adulthood. Spermatogenesis originates from SSCs that produce daughter germ cells to undergo sequential, synchronized, sequential, and extensive differentiation processes, eventually results in the formation of sperm. In female mice, PGCs enter the first meiosis process at E13.5 and arrest at the first meiosis. After birth, primordial follicles are formed (Spradling et al., 2011; Kanatsu-Shinohara and Shinohara, 2013). These follicles develop into primary, secondary, and antral follicles (McGee and Hsueh, 2000; Edson et al., 2009) and oocytes gradually acquire a gynogenetic epigenome (Lucifero et al., 2002; Kobayashi et al., 2012). The estrus cycle begins at approximately 6 weeks, which results in completion of the first meiosis and the formation of secondary follicles.

Key events of PGC specialization include partial reacquisition of the pluripotent gene network, such as the expression of *Sox2*, a master regulator of pluripotency, but the majority of PGCs, the gradual acquisition of PGC-specific genes, such as the expression of *Nanos3*, *Prdm1*, *Dppa3*, and *Prdm14*, and the differential repression of somatic mesodermal programs, particularly the repression of the *Hox* gene family, in the presence of their constant expression in neighboring somatic cells (Saitou et al., 2002; Seki et al., 2005; Yabuta et al., 2006). In mice, *Blimp1*, *Prdm14* (PR/SET domain 14), and *Tcfap2c* (also termed *AP-2*γ) are key genes needed for these events. BMP4 induces the expression of *Prdm1* and *Prdm14* (Lawson et al., 1999). *Prdm1* promotes the appropriate upregulation of many PGC-specific genes with high accuracy and inhibits almost all somatic genes (Saitou et al., 2005). *Prdm14-*involved PGC specification is a *Prdm1*-independent mechanism and *Prdm14* is involved in the reacquisition of potential pluripotency and epigenetic reprogramming (Saitou et al., 2005; Yamaji et al., 2008). *Tcfap2c*, which is expressed at E7.25-12.5, is a putative downstream factor of *Prdm1* and an effector molecule of *Prdm1* activity (Kurimoto et al., 2008). This gene inhibits mesodermal differentiation, downregulates *Hox* family genes, and represses the expression of *T (Brachyury)*, which is a marker of the mesoderm for the maintenance of PGCs (Weber et al., 2010). TCFAP2C is expressed at 12–37 weeks in human embryos, PGCs, and gonocytes (Pauls et al., 2005). The expression pattern is consistent with that found in mice, which suggests that TCFAP2C may have a conserved function in mice and humans (Weber et al., 2010).

Gametogenesis in humans is similar to that in mice, but species-depending variations in developmental timing and some molecular mechanisms have been observed. After the implantation of blastocysts, at approximately 2 weeks (Tang et al., 2015; Wen and Tang, 2019), human germ cells are specified as human PGCs (hPGCs) in the posterior epiblast that express the key transcription factors (TFs): SOX17, TFAP2C, and BLIMP1. However, the origin of hPGCs remains unknown, and the origin may be the amnion or the epiblast (Sasaki et al., 2016; Kobayashi et al., 2017). At approximately 5 weeks, these cells migrate to and colonize the embryonic gonads and initiate differentiation into gonocytes or oogonia in embryonic gonads (Baker, 1963; Fukuda et al., 1975). At 6–8 weeks, hPGCs undergo sexual differentiation and aggregate with gonadal somatic cells. During germ cell development, epigenetic reprogramming occurs by 10 weeks and includes imprint erasure, genome-wide DNA demethylation, and X chromatin reactivation (Gkountela et al., 2015; Guo et al., 2015; Tang et al., 2015). In the males, gonocytes differentiate into fetal spermatogonia during the embryonic period and then into spermatogonia at around birth. And after puberty, spermatogonia differentiate into sperm. Oogonia differentiate into oocytes, start meiosis and form primordial follicles with granulosa cells during the embryonic period. At birth, there are approximately 300,000 primordial follicles (Tomaselli et al., 2011). Where after puberty, only approximately 30,000 follicles survive and the ovary enters the ovarian cycle. A group of antral follicles go through recruitment and selection. Only one follicle develops first to become the dominant follicle, and this dominant follicle then completes ovulation.

The molecular mechanisms of mouse PGC (mPGC)and hPGC specialization are different. For hPGCs, SOX17, a critical transcription factor of the endoderm, is the key regulator and one of the most upstream TFs (Irie et al., 2015). *SOX17* promotes associated gene expression and mesoderm development and is the upstream of *BLIMP1* in hPGCs. However, *SOX17* is transiently upregulated upon mPGC specification, but its expression decreases as early as E7.5 and appears dispensable in mPGCs (Kurimoto et al., 2008). In addition, *SOX2* expression is regained during mPGC specification, but its expression is downregulated in hPGCs. Some researchers have reported that hPGC specification is concomitant with upregulation of the somatic mesodermal program, although at a markedly slighter level, and includes downregulation of a program for “neuron development” (Sasaki et al., 2015). The role of *BLIMP1* also different between hPGCs and mPGCs. *BLIMP1* inhibits the somatic mesodermal program in mPGCs and plays a role in inhibiting neuron development in hPGCs (Sasaki et al., 2015). The differences in germ cell development caused by the distance between human and mouse species make the study of human germ cell development using mouse models insufficient. However, the small number of hPGCs, technical problems and ethical issues drive scientists to seek new methods.

**FIGURE 1:**
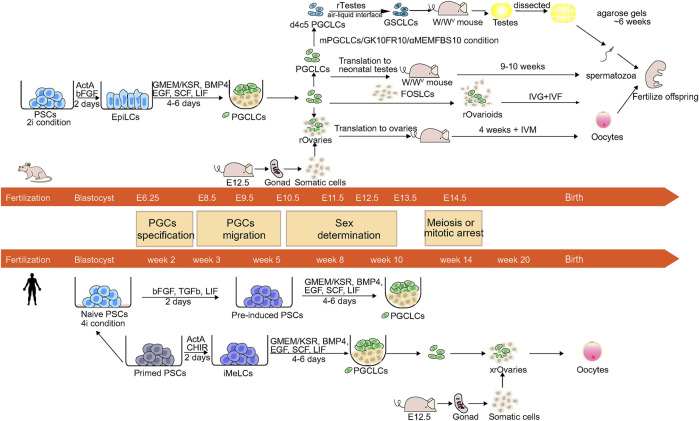
Schematic of the germ cell development and reconstitution of mPGCLCs and hPGCLCs *in vitro*. The center is the image shows developmental stages and the timeline of mouse and human germ cell development. PGCs undergo specification, migration, sex determination, and meiosis or mitotic arrest before birth. The upper and lower panels show the induction of mPGCLCs and hPGCLCs.

In recent years, many studies have elucidated the processes and mechanisms of mouse and human germ cell development. In many cases, the generation of human *in vitro* induced gametes mimics the methods of the generation of mouse *in vitro* induced gametes. Based on these findings, some research groups have successfully reconstituted the germ cell specification pathway and generating PGC-like cells (PGCLCs) and produced fertile offspring through the use of PSCs (Figure 1).

## *In Vitro* Induced Gametes Using Pluripotent Stem Cells

Many studies have used PSCs to reconstruct germ cell development in mice and humans. Based on abundant knowledge of mouse gametogenesis, some *in vitro* reconstructed mouse gametogenesis models have been established, and these models can be modified to adapt to human germ cell development and reconstruct human gametogenesis. We mainly focused on the methods that differentiation of embryonic stem cells (ESCs) or induced pluripotent stem cells (iPSCs) into PGCLCs and subsequently the induction of PGCLCs into gametes. To analyze the properties and origin of the germ cell lineage *in vivo* and establish a culture system to efficiently generate induced gametes with temporally coordinated gene expression *in vitro*, noninvasive fluorescent protein labeling systems are often used. BVSC system, which is one of the most widely used systems (Ohinata et al., 2008), includes membrane-targeted Venus (mVenus) controlled by *Blimp1* and Enhanced Cyan Fluorescent Protein (ECFP) controlled by *Stella*. The system contains two fluorescent markers and is capable of marking PGC precursors at E6.25 (Ohinata et al., 2005) and migrating PGCs E8.5. PGCs precursors are Prdm1-positive and migrating PGCs are Prdm1-and Stella-positive (Saitou et al., 2002; Ohinata et al., 2008).

In the past, some researchers have induced the differentiation of mouse and human ESCs (mESCs and hESCs, respectively) to gametes or PGCs, but the efficiency was too low (Daley, 2007; Saitou and Yamaji, 2010). Mouse Epiblast cells at E5.5-6.0 are able to express key PGC genes: *Prdm1* and *Prdm14* under BMP4 stimulation (Ohinata et al., 2009). Therefore, the acquisition of E5.5-6.0 mouse Epiblast cells or similar cell lines may enable the *in vitro* reconstruction of embryogenic development and the acquisition of PGCs or gametes (Hayashi et al., 2011). Two pluripotent stem cell lines in mice, mouse ESCs/iPSCs and mouse Epiblast Stem cells (mEpiSCs) correspond to the E3.5-4.5 inner cell mass (ICM) and E5.5-6.5 epiblasts, respectively (Evans and Kaufman, 1981; Brons et al., 2007; Tesar et al., 2007). Although EpiSCs can express *Prdm1*, the percentage of *Prdm1*-positive cells remains low under BMP4 stimulation (Hayashi and Surani, 2009). Thus, Hayashi et al. used the BVSC system to trace the production of germlines and developed a method to produce mouse PGCLCs (mPGCLCs). These researchers induced mESCs and miPSCs into pregastrulating E5.75 epiblast-like cells (EpiLCs), which can be stimulated by BMP4 to form germ lines. The mVenus fluorescent marker shows that d2 EpiLCs are *Blimp1*-positive, and the induction time is very short. Long-term culture transforms mESCs/miPSCs into EpiSCs, which indicates that epiblasts form germinal cells within a very short period, and this process is strictly controlled within E5.5-6.0. EpiLCs are then induced to differentiate into mPGCLCs. BVSC-positive foci appeared from day 4 (∼13.5%) to day 6 (∼7.2%) of culture under full induction conditions, and the cell population expanded during this period. Transcriptome and epigenome analysis showed that key events of mPGCLC specialization occurred during the induction of mPGCLCs. The key events consist of reacquisition of some pluripotent gene networks, such as the upregulation of *Sox2* and *Nanog*; the expression of PGC-specific genes, such as the *Prdm1, Prdm14, Tcfap2c, Nanos3,* and *Stella*; the inhibition of somatic mesodermal processes, such as the repression of *Hox* family genes; epigenetic reprogramming, such as the downregulation of *Dnmt3a/3b* genes, and the erasure of DNA methylation (Yabuta et al., 2006). A comparison of global transcriptome patterns, revealed that d2 mEpiLCs and d6 mPGCLCs have a transcriptome similar to that of E5.75 epiblasts in transcriptome and an epigenome similar to those of E9.5 migratory mPGCs respectively. Therefore, the induction pathway of mPGCLCs almost recapitulates that of mPGC specification *in vivo* (Hayashi et al., 2011). In addition, these researchers found that by combining Integrin-β3 and SSEA1 as markers, PGCLCs with spermatogenic capacity can be isolated from tumorigenic undifferentiated cells (Hayashi et al., 2011).

To evaluate the function of mPGCLCs, scientists have performed a series of tests. For males, mPGCLCs (XY) were transplanted into the seminiferous tubules of W/W^v^ neonatal mice lacking endogenous germ cells (Chuma et al., 2005). Notably, the results that these seminiferous tubules contained dark central sections corresponding to spermiation and were markedly thicker than those without spermatogenesis showed mPGCLCs contributed to spermatogenesis (Chuma et al., 2005; Ohinata et al., 2009). Then the spermatozoa were segregated from reconstituted testes and injected into oocytes by intracytoplasmic sperm injection (ICSI). The resultant zygotes developed normally, they then were transferred to foster mothers. And these embryos successfully developed into healthy offspring with normal placentas and appropriate imprinting patterns (Hayashi et al., 2011).

Researchers have also tested the function of mPGCLCs in females and explored the subsequent gametogenesis *in vitro* (Hayashi et al., 2011). When mPGCLCs (XX) have aggregated with E12.5 female gonadal somatic cells in reconstituted ovaries (rOvaries), they underwent epigenetic reprogramming, such as X chromatin reactivation and imprint erasure, and exhibited meiotic potential. After the transplantation of rOvaries under the mouse ovarian bursa, mPGCLCs mature into germinal vesicle-stage oocytes. The oocytes then underwent *in vitro* maturation (IVM) and fertilization (IVF) contributed to fertile offspring (5/127, ∼3.9%). These researchers also obtained healthy offspring from embryos derived from natural oocytes (7/55, ∼12.7%) and E12.5 PGCs (13/75, ∼17.3%) (Hayashi et al., 2012).

To obtain a long-term model of *in vitro* gametogenesis, Hayashi et al. reconstituted the complete process of oogenesis *in vitro* (Hikabe et al., 2016). These researchers induced miPSCs or mESCs to differentiate into mPGCLCs by the same method as that used in their previous research (Hayashi et al., 2011). They divided the culture systems from mPGCLCs to mature oocytes into three stages: *in vitro* differentiation (IVDi), *in vitro* growth (IVG), and IVM. In the IVDi stage, they got rOvaries were obtained as in their previous culture system (Hayashi et al., 2012), but the difference was that rOvaries were cultured *in vitro* for 3 weeks. SC (ECFP controlled by Stella)-positive primary oocytes and Foxl2-positive (a granulosa cell functional marker) granulosa cells were observed, which suggests the formation of a primary follicular structure. The second stage was the IVG stage: the follicles were separated from the rOvaries and after 11 days of culture, the primary oocytes grew into germinal vesicle (GV) oocytes. In the third stage, GV oocytes were transferred to IVM culture condition, 28.9% of oocytes could extrude the first polar body and enter the second meiosis stage. To test the function of metaphase II (MII) oocytes, MII oocytes were fertilized with natural sperm in IVF and these zygotes developed into healthy offspring. Although the success rate of obtaining pups from *in vitro* induced oocytes was 3.5%, which was lower than that of obtaining pups from natural eggs derived from *in vivo*, the study successfully reconstituted the entire cycle of oogenesis *in vitro* and produced healthy offspring.

Zhou et al. invented an induction system (Zhou et al., 2016) to achieve *in vitro* male gametogenesis in mice. mESCs were differentiated into d6 mPGCLCs according to Hayashi et al. (2011). Zhou et al. used an adaptation of this method to generate mPGCLCs. To reconstitute the *in vivo* environment, these researchers cocultured mPGCLCs with early postnatal testicular cells, and exposed these cells to retinoic acid (RA) (Menke et al., 2003; Bowles et al., 2006; Zhou et al., 2008), BMPs, and activin A (Hu et al., 2004; Puglisi et al., 2004). After 6 days of culture, PGCLCs exited the state of PGCs/SSCs, initiated meiosis, and integrated into a colony of multiple testicular cell types. Subsequently, these cells were exposed to follicle-stimulating hormone (FSH), bovine pituitary extract (BPE), and testosterone (O'Shaughnessy, 2014), and after 8 days of culture, haploid spermatid-like cells (SLCs) were generated. The sorted SLCs could form fertile offspring by ICSI. The culture system can be used to explore the process of gametogenesis and the function of morphogens and hormones in gametogenesis. And these results need to be replicated by independent groups. But the success rates of ICSI to obtain live births with spermatids derived from mESCs were 2.8% with spermatids derived from mESCs and 9% with round spermatids *in vivo*. This low rate of pregnancy outcome possibly reflects the inappropriate epigenetic status of the spermatids obtained in culture (Bhartiya et al., 2016). In addition to the BVSC system, the SGPD system includes EGFP-fused retinoic acid gene 8 (*Stra8*) (marking early-stage spermatogonia through preleptotene-stage spermatocytes) (Li et al., 2007), and DsRed-fused protamine 1 (*Prm1*) (marking postmeiotic spermatids). The combination of BVSC and SGPD systems allows the monitoring of PGC specialization and spermatogenesis, and the screening of cytokines and hormones at appropriate time points (Zhou et al., 2008; Zhou et al., 2016).

d4 mPGCLCs induced from mouse PSCs (mPSCs) can differentiate into SLCs when aggregated with E12.5 embryonic testicular somatic cells. These cells can propagate as germline stem cell-like cells (GSCLCs) (Ishikura et al., 2016). Germline stem cells (GSCs) are able to both differentiate into mature cell types (gamete) and exhibit self-renewal properties to maintain an undifferentiated state (Kanatsu-Shinohara et al., 2003). GSCs could produce normal spermatozoa and offspring after transplanted into infertile mice (Kanatsu-Shinohara et al., 2005). However, the differentiation process of GSCLCs is less efficient and delayed compared with the *in vivo* process (Ishikura et al., 2016). Ishikura et al. improved the differentiation scheme by exploring different starting materials for mPGCLCs in different culture periods and various culture conditions. They also used a BVSC system plus RFP under the control of Ddx4. Ddx4 begins to be expressed in gonocytes and showed increased expression after male germ-cell specification. d4c5 mPGCLCs [d4 mPGCLCs cultured with forskolin (10 μM), rolipram (10 μM), and cyclosporin A (5 μM) (FR10Cs5) for 5 days] aggregated with E12.5 embryonic testicular somatic cells (rTestes) exhibited the best outcome in generating SLCs according to the number (∼450) and ratio (∼90%) of the VR+ cells after 7 days of air-liquid interface culture on the membrane (Ishikura et al., 2021). Moreover, these SLCs can generate sperm upon transplantation into testes and *in vitro* culture of testis transplants (Ishikura et al., 2021).

However, obtaining embryonic gonadal somatic cells, particularly human embryonic gonadal somatic cells is difficult, in addition, these methods of using embryonic gonadal somatic cells cannot easily maintain homogeneity, and their large-scale application is difficult. In addition, the *in vitro* transplantation of gametes into the human body for development and testing is difficult, which prevents research on developing human *in vitro* induced gamete systems. At the same time, the connection between germ cells and somatic cells is critical to the development of germ cells. For example, the maturation of germ cells needs signals and nutrients from surrounding somatic cells to enter meiosis (Li and Albertini, 2013). Therefore, the studies on the cellular interaction and paracrine signal provide the possibility to reconstruct mature gametes induced by stem cells *in vitro*.

Recently, the generation of fetal ovarian somatic cell-like cells (FOSLCs) derived from mESCs have brought new hope (Yoshino et al., 2021). FOSLCs express *Nr5a1*, a representative marker gene of gonadal somatic cells (Luo et al., 1994; Stevant et al., 2019), and a transcriptomic analysis has shown that the transcription pattern and cellular composition of FOSLCs were similar to that of E12.5 embryonic ovarian cells. After the aggregation of FOSLCs with mPGCLCs, the mPGCLCs generated functional oocytes in the reaggregates, termed reconstituted ovarioids (rOvarioids). FOSLCs are sufficient to support the development of germ cell progenitors into functional oocytes capable of producing fertile offspring. The ability to produce and assemble the crucial components needed for oocyte development provides a model system for studying the later events of oocyte development, which may have implications for clinical applications (Yang and Ng, 2021). However, the efficiency of producing oocytes with FOSLCs is lower than that with gonadal somatic cells. For example, the number of oocytes in rOvarioids with FOSLCs was reduced to 67.4% of that in ovarioids with E12.5 gonadal somatic cells in C57BL/6J mice. Therefore, FOSLCs have great potential for optimization, such as improving the oocytes’ production efficiency of rOvarioids, improving methods to purify FOSLCs and further identifying the molecular mechanism of FOSLCs. This process could be attempted in other animals, such as non-human primates (NHP) to extrapolate the possibility of *in vitro* induced gametes in humans (Yoshino et al., 2021).

A novel cell line of pluripotent stem cells from adult gonads, termed very small embryonic-like stem cells (VSELs) in humans, mice and other mammals, is a possible source for obtaining gametes *in vitro* (Anand et al., 2015; Bhartiya et al., 2017). Parte et al. reported that adult perimenopausal ovarian VSELs express *Stella* and *Fragilis* (specific markers for PGCS), which suggests that VSELs are indeed PGCs and are SSCs in the testis and ovarian stem cells (OSCs) in the ovary (Parte et al., 2014). VSELs are relatively more mature than ESCs and iPSCs and can spontaneously differentiate into sperm and oocytes. VSELs are pluripotent but relatively quiescent due to epigenetic modifications of parentally imprinted genes (Bhartiya et al., 2014). As result of their quiescent state, these cells can survive after organ damage and chemotherapy in mice (Kurkure et al., 2015; Sriraman et al., 2015) and humans (Kurkure et al., 2015), which allows their use in regenerative therapy and fertility restoration in chemotherapy patients. It has been reported that VSELs produce SSC-like cells through asymmetric cell division and then form cell aggregates through symmetrical division (Patel and Bhartiya, 2016). The obtained cells have been cultured with Sertoli cells and stimulated with FSH for 3 weeks to obtain various stages of spermatogenesis (Anand et al., 2015). However, due to the limitations of human reproductive ethics, researchers have not conducted reproductive tests with the derived sperm but detected SSC markers and meiosis markers. However, SSCs have no specific markers, and the function of SSCs can only be detected by transplantation assays. Therefore, although they show the biological characteristics of stem cells, the true identity of these cells remains questionable. However, some studies can reflect the capability of VSELs to undergo gametogenesis. Anand et al. cultured all mouse testicular cells *in vitro* after chemical ablation and found that these cells could undergo spermatogenesis (Anand et al., 2015). VSELs and SSCs proliferated, underwent spermatogenesis, and meiosis and differentiated into sperm, as indicated by the expression of transcripts specific for different stages of spermatogenesis (Anand et al., 2015). Although it is be possible that only a few SSCs formed sperm *in vitro* rather than VSELs (Zhou et al., 2016), the fact that a few surviving SSCs can also undergo the spermatogenesis may also offer hope for assisted reproductive treatment. In contrast, due to the sophisticated isolation methods that are needed, confirmation criteria and the low availability of ovarian tissues, the usage of VSELs for fertility therapy at a large scale is limited, and the proper exploration of this possibility needs further research (Bharti et al., 2020). There is much debate about VSELs, and more studies are needed to further verify the potential of VSELs and their ability to differentiate into gametes.

Many studies have attempted to generate human germ cells, and the strategies are based on the generation of mouse germ cells. For example, the induction of human PSCs (hPSCs) to human PGCLCs (hPGCLCs) mimics the pathway for the induction of mPGCLCs. However, some differences between mPSCs and hPSCs must be considered and modified to induce hPGCLCs. Moreover, the most reliable method for identifying the induced germ cells is the production of fertile offspring. Mouse induced germ cell lines can be identified through reproduction and the production of fertile offspring. However, according to the 2021 ISSCR (International Society for Stem Cell Research) Guidelines for the Field of Stem Cell Research and Regenerative Medicine (Lovell-Badge et al., 2021), the use of gametes differentiated from human stem cells for reproduction is not allowed and currently thought to be unsafe because of the unsafe approaches or unresolved ethical issues. Therefore, it is necessary to seek other methods for verification, such as morphology, spindle, specific marker detection, multiomics analysis, and *in vitro* reconstruction of gonads.

According to the methods for the generation of mPGCLCs, pregastrulating epiblast-like cells should be first derived and subsequently differentiated into PGCLCs. The states of hESCs and human iPSCs (hiPSCs) are similar to the state of mEpiSCs (Brons et al., 2007; Tesar et al., 2007) and these cells are inappropriate for the generation of PGCLCs. Therefore, the first step is to induce hESCs or hiPSCs to become pregastrulating epiblast-like cells. Two groups have successfully generated hPGCLCs successfully. Irie et al. (2015) reported that hESCs/hiPSCs cultured in “4i” medium (inhibitors four kinases: MAPK, GSK3, p38, c-Jun N-terminal kinase) are in a naïve state of human pluripotency (Gafni et al., 2013) and are efficiently induced into hPGCLCs (∼27% of NANOS3/TNAP, a PGC gene and a PGC and pluripotency markers respectively, double-positive hPGCLCs) with using a procedure similar to induction of mPGCLCs. Another group cultured hiPSCs under feeder-free conditions (the primed state of pluripotency), and hiPSCs first differentiated into incipient mesoderm-like cells (iMeLCs) and then into hPGCLCs (∼30–40% of BTAG-positive cells at day 2 and ∼60% of BTAG-positive cells at day 4) based on a procedure similar to inducing mPGCLCs (Sasaki et al., 2015). The hPGCLCs in the two protocols exhibit gene expression similar to week 7 hPGCs but do not exhibit the expression of *DDX4* and *DAZL* (markers of germ cells), which shows that the hPGCLCs correspond to hPGCs at an early stage.

Yamashiro, C. et al. took a major step forward successfully in human vitro gametogenesis by coculturing hPGCLCs with mouse embryonic ovarian somatic cells to generate xenogeneic reconstituted ovaries (xrOvaries), a type of organoid, which were cultured *in vitro* for 4 months to produce oogonia (Yamashiro et al., 2018). The entry of hPGCLCs into gametogenesis *in vitro* is possible due to the successes of *in vitro* gametogenesis in mice by the coculture of mPGCLCs with mouse embryonic somatic gonadal cells *in vitro* or their transplantation into the corresponding position *in vivo* (Hayashi et al., 2011; Hayashi et al., 2012; Hikabe et al., 2016; Ishikura et al., 2016; Luo et al., 2021). However, human embryonic somatic gonadal cells cannot easily obtain due to ethical and technical difficulties, which also prevents the verification of the ability of hPGCLCs to enter the later stage of germ cell development. Therefore, the use of xenogeneic embryonic gonadal cells appears to be a possible solution. Yamashiro, C. et al. traced the process of cell lineage changes with fluorescent proteins. The *BLIMP1*-tdtomato, *TFAP2C*-EGFP (BTAG) system can mark hPGCLCs, and can also label gonocytes or oogonia at least until week 10 (Gkountela et al., 2015; Guo et al., 2015; Tang et al., 2015; Bharti et al., 2020). The BTAG-positive d6 hPGCLCs have been isolated to generate xrOvaries. Within 21–77 days of xrOvaries, the number of BTAG-positive cells was markedly decreased (from ∼2,000 to ∼500), and only a small number of cells survived. The expression of germ cell genes, *DAZL,* and *DDX4*, and meiosis genes suggested the cells developed further. These results have prompted the researchers to continue culturing xrOvaries, and female hPGCLCs have been cultured for up to 4 months. To mark the cell lineage at later stages, *DDX4* [also known as human VASA homolog (hVH)] has been used instead of *BLIMP1* to control tdTomato expression (VT) and *TFAP2C* has been used to control EGFP expression, to form the AGVT system. At 120 days, AGVT-positive cells differentiated into AG-negative VT-positive cells. An analysis of the expression of key genes, such as PGC genes, pluripotency genes, germ cell genes, and meiosis-related genes, suggested that the cells in extended culture continued to develop forward, but that meiosis recombination had not yet started. A transcriptome analysis has also shown that cells at 77 and 120 days may correspond to gonocytes or oogonia at 7 and 9 weeks, respectively. By further examining the genes of the germ cells of human fetal germ cells in females at different stages, the stage of these cells at 120 days were found to correspond to the stages of oogonia and RA responses. Epigenetic reprogramming events have also been analyzed. The derived oogonia display hallmarks of epigenetic reprogramming, including genome-wide DNA demethylation, imprint erasure, extinguishment of aberrant DNA methylation in hiPSCs, a progressive and partial demethylation and reactivation in the inactive X chromosome (Guo et al., 2015; Tang et al., 2015; Patel et al., 2017).

Mouse granulosa cells provide a permissive environment for the development of hPGCLCs into oogonia, which suggests that processes of germ cell specialization and the signaling pathways are relatively conserved, and these finding provide certain references for the development of human germ cells during later gamete development. Because male mPGCLCs can differentiate into SLCs after the generation of mouse rTestes, it is possible that male hPGCLCs can enter meiosis through a similar method. The construction of mouse FOSLCs may also be applied to the induction of human embryonic gonadal cells, which provides a possible solution to the problems of obtaining human embryonic germ cells and a platform to study the related molecular mechanisms. Further optimization and improvement are needed in the future, and it is possible to obtain mature oocytes and sperm.

PGCs differentiate into gametes, which fertilize to produce totipotent embryos through fertilization. Therefore, PGCs are the source of totipotency. Gametogenesis involves specific processes including global epigenetic reprogramming, cell interaction, and migration (Richardson and Lehmann, 2010). The study of these specific processes will also be helpful in other areas. For example, research on PGC migration provides a useful system for obtaining a pathological and physiological understanding of a group of individually migrating cells *in vivo*. The processes of PGC specification and migration are closely related to epigenetic reprogramming, whereas epigenetics does not change genetic information but heritably changes gene function. Inappropriate epigenetic changes may reflect abnormal developmental processes. The study of germ cell genesis is also of great significance to obtaining an understanding of epigenetic mechanisms.

The development of these methods has greatly improved the understanding of the mechanisms and pathological processes of human gamete development, although some defects exist and *in vitro* gametogenesis exhibits a delay compared with *in vivo* gametogenesis using some methods. Understanding the mechanisms of gamete development, in turn, promotes the development and improvement of induction systems. The methods for organoid construction methods using reconstructed gonads help enter the later stages of gamete development, including oogonia, SLCs and oocytes, among others, greatly contributing to *in vitro* gametogenesis. The application of new organoid technologies may provide new ideas for the development of organoid systems, for example, the application of microfluidics. However, the ethical issues of germ cells induced *in vitro* need to be treated with great caution. According to the 2021 ISSCR guidelines, *in vitro* gametogenesis without the fertilization or generation of embryos is not typically reviewed by a specialized oversight process, but the use of gametes differentiated from human stem cells for reproduction is not allowed (Lovell-Badge et al., 2021). However, research on human *in vitro* gametogenesis is still needed because *in vitro* gametogenesis will serve as a platform not only to advance the germ cell biology foundation but also to develop innovative medical applications. Such research on other animals, particularly NHPs, is also a great idea.

## Application of Embryoids

In mice, sperm fertilize the oocyte to form the diploid zygote and initiate embryogenesis, and the zygote then undergoes a series of mitosis to form blastomeres. The minor wave of embryonic genome activation (EGA) occurs at the zygote and early 2-cell stages. The major EGA mainly occurs at the late 2-cell stage. Between the 8- and 16-cell stages, the blastomere undergoes compaction and exhibits polarity. At approximately E3, the embryo undergoes cavitation and first cell lineage separation, which forms the ICM and trophectoderm (TE). The cavity marks formation of the blastocyst. At E4.5, the ICM of the late blastocyst develops into epiblasts and primitive endoderm (PrE). The late blastocyst implants into the uterus. Between E6.5 and −7.5, the embryo undergoes gastrulation. Human embryogenesis is similar to mouse embryogenesis, but there are some differences. In humans, minor EGA occurs at the 2-cell stage, and major EGA occurs between the 4- and 8-cell stages. Between E5 and -6, the cavity forms and marks formation of the early blastocyst consisting of the ICM and TE. At the late blastocyst stage, the ICM is separated into epiblasts and hypoblasts (or PrEs). Epiblasts form three germ layers of the embryo, and hypoblast develop into the yolk sac. At approximately E7, the blastocyst implants into the uterine endometrium. At approximately E14, the PS appears and is located in the epiblast. The embryo initiates gastrulation (Wamaitha and Niakan, 2018).

Our understanding of human embryogenesis was obtained from the human IVF and samples from the Carnegie Institution. IVF, in association with single-cell multiomics analysis, can demonstrate the main events and underlying molecular mechanisms in human preimplantation development. Based on the contributions of the Carnegie Institution, researchers have basically uncovered early human postimplantation morphogenesis. In contrast, with the application of mouse and NHP models, researchers have obtained much knowledge of human embryo development. However, species differences and limitation of human nature embryos indicate the need for new strategies for studying embryonic development. Similar to gametogenesis, *in vitro* embryogenesis has grabbed much attention and human *in vitro* embryogenesis is based on systems in mice. Organoids that improve the generation of model systems with similar cell types and conditions are regarded as great research tools. Researchers have generated embryoids, blastoids, and gastruloids *in vitro* (Table 1), thus, it is possible to uncover complex embryonical events and establish key developmental principles in a detailed and highly quantitative manner.

**TABLE 1:** Embryoids and stem cell models.

Types	Initial cells	Characteristics	Methods	References
Embryoid bodies (EBs)	Mouse clonal pluripotent	Identical to intraperitoneal teratocarcinomas, which	Cultured without feeder cells	Martin and Evans, (1975)
	Teratocarcinoma cells	Consist of an inner core of embryonal carcinoma cells surrounded by a layer of endodermal cells		
Embryoid bodies (EBs)	HESCs	Comprise three embryonic germ layers	Suspension, without LIF, and bFGF	Itskovitz-Eldor et al. (2000)
Blastoids	mESCs, mTSCs	Structures that morphologically and transcriptionally resemble embryonic Day 3.5 blastocysts	Sequential aggregation of ESCs and TSCs in microwells	Rivron et al. (2018)
Blastoids	mEPSCs (LCDM-EPSCs and Liu’s EPSCs)	Mouse blastocyst-like structures in terms of morphological and can be implanted to induce decidua formation	3D differentiation system with 2:1:1 mixture of KSOM, TSC basal and N2B27 basal medium to support the growth both ESC and TSC aggregates	Li et al. (2019)
Blastoids	mEPSCs, mTSCs	All three blastocyst lineages in blastocyst-like structures	Compacted amorphous EPSC aggregates plus TSCs in microwell-based system using sequential changes of different media under hypoxic culture conditions	Sozen et al. (2019)
Blastocyst-like cyst (iBLs)	MPSCs	A blastocoel-like cavity and outer cells expressing trophectoderm lineage markers and inner cells expressing implantation-competent pluripotency markers	Combination of LIF, BMP4, LPA, and AA converts mouse primed PSCs into iBLC PCs and produces iBLCs	Kime et al. (2019)
Human-blastoids	naïve hPSCs	Resemble human blastocysts in terms of morphology, size, cell number, and composition and allocation of different cell lineages	3D culture coupled with sequential treatment of WIBR3 cells with HT or TH method	Yu et al. (2021)
Blastoids	naïve hPSCs	Consist of three tissue layers displaying exclusive lineage markers, which mimics the natural blastocyst	brief induction of TE by modulating PD + A83 treatment	Yanagida et al. (2021)
iBlastoids	HiPSCs	Model the overall architecture and transcriptomic profiles of blastocysts and, ICM-like, Epi-like and PrE-like structures and cells	Transfer of a reprogramed and mixed cell population in 3D culture and a medium that sustains the early developmental signatures	Liu et al. (2021)
Human EPS-blastoids	HEPSCs	Resemble human blastocysts in terms of morphology, the expression of markers specific for 3 cell lineages, and global transcriptome signatures	3D, two-step differentiation protocol	Fan et al. (2021)
Gastruloids	mESCs	Multicellular models of a gastrulating embryo	Small aggregates of mESCs, of approximately 300 cells	van den Brink et al. (2014)
Gastruloids	hESCs	Recapitulate the embryonic arrangement of the mammalian germ layers	Colonies of hESCs grown on micropatterned substrate and differentiated with BMP4	Etoc et al. (2016)
Gastrulation-like	hESCs	Colonies reproducibly differentiated to an outer trophectoderm-like ring, an inner ectodermal circle and a ring of mesendoderm expressing primitive-streak markers in between	Geometric confinement of colonies and response to BMP4	Warmflash et al. (2014)
Human pregastrulating epiblast	hESCs	Display a similar size range, morphology and tissue polarity that match those of epiblasts of *in vitro* attached Day 10 human embryos	Dispersedly dissociated hESCs as single cells in Matrigel and in culture under pluripotency conditions	Simunovic et al. (2019)

Embryonic bodies are disorganized 3D clusters of pluripotent or differentiated cells that are not considered embryoids. Embryoids are more organized embryoid bodies due to the correct topology, resulting from multiple cell types representing a defined stage of the embryo or cell polarization induced by the extracellular matrix (ECM) in the surrounding medium. Blastoid constitute a typical example of embryoid.

### Generation of Mouse and Human Blastoids and Their Applications and Shortcomings

There is little information about blastocyst development in human, for example, the interaction of ICM and the = TE that are generated by the first lineage commitment and morphogenesis in the embryogenesis. However, due to the temporal overlap and technical limitations, the exploration of these unknow questions is difficult. Numerous studies on mouse early embryonic development have uncovered much information, but human models are needed due to species differences. Similar to *in vitro* gametogenesis, mouse models followed by human models have been generated.

Recently, Rivron et al. generated *in vitro* structures that morphologically and transcriptionally resemble embryonic day 3.5 (E3.5) blastocysts in mice, and these structures are termed blastoids (Rivron et al., 2018). These researchers aggregated mESCs and added mouse trophoblast stem cells (mTSCs) and these cells were sequentially seeded under 3D suspension conditions. The aggregates formed blastoids (70% with the combination of 8 ESCs and 20 TSCs), which act like mouse blastocysts and are formed when the signals of embryonic cells induce trophoblast development. Blastoids are used as models to investigate the signaling and molecular mechanisms of TE induction in embryonic cells. These researchers found that embryonic cells regulate TE proliferation and self-renewal through STAT and MAPK pathways, and that the BMP4, Nodal, and WNT pathways regulate TE epithelial morphogenesis. In addition, the research contents including the transcriptome, signaling pathway, and exploring embryonic developmental mechanisms using blastoids can be used as a paradigm of blastoid.

Other types of blastoids have been generated *in vitro* from different stem cell types and through different combinations of inhibitors and growth factors. Recently, two research groups used D-EPSCs (Deng laboratory’s expanded pluripotent stem cells) (Yang et al., 2017) to generate mouse blastoids. One type was blastoids generated only by EPSCs from the Belmonte group (EPS-blastoids) (∼15% of the cell aggregates exhibit typical blastocyst-like morphology with the seeding of ∼5 cells per microwell) (Li et al., 2019) and the other is produced by EPSCs in combination with TSCs from the Zernicka-Goetz group (ZG-blastoids) (15.17% of cystic structure formation rate under 5% O_2_) (Sozen et al., 2019). In ZG-blastoids, EPSCs and TSCs self-organized into blastocyst-like structures with all three embryonic and extraembryonic lineages. In terms of morphology and transcriptome, ZG-blastoids show embryonic-abembryonic axes and primitive endoderm differentiation and can start the transition from preimplantation to postimplantation to result in egg cylinder morphology *in vitro* (Sozen et al., 2019). EPS-blastoids resemble blastocysts in morphology and cell lineage allocation and recapitulate key developmental events, including compaction, polarization, and paternal X chromosome inactivation during the first preimplantation for the first time. Most notably, these blastoids were capable of implanting *in utero* and of being generated only by a single cell type, which may enable us to obtain more genetic and epigenetic information (Li et al., 2019). In addition, a research group induced self-organizing 3D blastocyst-like cysts (iBLCs) from mPSCs(primed pluripotent state). These cells harbor a blastocoel-like cavity (usually ∼5–50% of clusters formed a blastocoel-like cavity) and the blastocyst-like cysts consist of inner cells expressing pluripotency markers and outer cells expressing trophectoderm lineage markers. When transplanted to pseudopregnant mouse uteruses, the cells undergo implantation, induction of decidualization, and growth (Kime et al., 2019).

Blastoids provide a unique platform for studying early embryogenesis and pave the way to creating viable synthetic embryos by cultured cells. These cells can be models for studying biological questions regarding preimplantation and early postimplantation embryogenesis and modeling diseases associated with early pregnancy. Moreover, mouse blastoids can serve as a framework for propelling the development of functional synthetic blastocysts, in not only mice but also other mammalian species, including humans.

In further analysis, both types of blastoid cells were aligned well with embryo cells, although at the gene regulatory level, but blastoids and blastocysts exhibit apparent differences in TE lineages, which indicates that the gene regulatory programs of developing blastocysts represented in blastoids derived from D-EPSCs cannot fully exhibit some features of natural blastocysts. A large proportion of the blastoid cells generated with only D-EPSCs showed expression of genes associated with postimplantation stage lineages and not cells of the blastocysts. Based on transcriptome data, only 6.7% of EPS-blastoid cells clustered close to the TE lineage. Sixty percent of EPS-blastoid cells consisted of two intermediate clusters that did not align with any blastocyst cells but instead were most closely similar to postimplantation-stage embryo cells. In ZG-blastoids, no detectable contribution of D-EPSCs to the TE lineage have been observed, and these cells could not incompetent to form blastoids in the absence of TSCs (Posfai et al., 2021).

Models of preimplantation human blastocysts were not previously available, and we have obtained much knowledge from mouse embryos and mouse EBs. However, given the significant differences between mice and humans, for instance, segregation of epiblasts and hypoblasts occurs after implantation in human embryos but not mouse embryos, human blastoids are alternative models to blastocysts for studying early human development.

Recently, several independent research groups have generated human blastoids *in vitro* using EPSCs, hESCs, iPSCs, or hiPSCs. Yu et al. generated human blastoids from naïve hPSCs that were cultured in 5i/L/A medium [a combination of five compounds, including inhibitors of MEK, GSK3, BRAF, ROCK, and SRC and leukemia inhibitory factor (LIF) supplemented with activin A] (Theunissen et al., 2014). These researchers obtained two types of differentiation media, hypoblast differentiation medium (HDM) consisting of N2B27 medium with a combination of FGF2, activin A and a WNT activator, and trophoblast differentiation medium (TDM) consisting of a 1:1 mixture of TSC medium and 5i/L/A medium These cells were treated with sequential HDM and TDM (12.8% of the aggregates’ exhibited structures with a cavity and contained the correct location of specific markers) or sequential TDM and HDM (9.4% of aggregates’ exhibited structures with a cavity and contained the correct location of specific markers) in a 3D system to promote cell differentiation into trophectoderm and hypoblasts (Yu et al., 2021). In addition, the researchers found that specific isozymes of protein kinase C (PKC) play a critical role in formation of the blastoid cavity (Yu et al., 2021). Liu et al. reprogrammed fibroblasts to produce a mixed cell population that contained epiblast-like, trophectoderm-like, and hypoblast-like cells with a well-established transcription factor cocktail including *OCT4, KLF4, SOX2* and *c-MYC.* Then, these researchers then transferred these reprogrammed cells into 3D culture system and formed 3D models of the human blastocyst, named iBlastoids (∼5.8–18% of the iBlastoids formation efficiency) (Liu et al., 2021). Fan et al. also developed a 3D two-step differentiation protocol to generate human blastoids, named EPS-blastoids. In addition, 53.5% of day 6 EPS-blastoids contained the correct pattern of ICM-(OCT4+) and TE-like (CK8+). Human EPS-blastoids resembled human blastocysts in terms of morphology, composition, allocation of three cell lineages and global transcriptome signatures at a single-cell resolution (Fan et al., 2021). More notably, further *in vitro* culturing of EPS-blastoids resulted in structures resembling early postimplantation embryos. Further applications require improvement, such as normality of the chromosome setup, epigenetic profile, and proper lineage development. Yanagida et al. produced human blastoids using naïve stem cells, which show high fidelity to human embryos (Yanagida et al., 2021). Although these blastoids are exciting models for studying early human development, many improvements are needed before they become a widely used research tool. For example, there are questions regarding the low efficiency and poor reproducibility of the blastoids across experiments, and cultured artifacts may not reflect real biological events. More induction protocols will be helpful for resolving these questions (Popovic et al., 2021).

### Establishment of Mouse and Human Gastruloids and Their Application and Perspectives

Gastrulation is a dynamic and well-orchestrated process of embryogenesis. During gastrulation, cells undergo dramatic morphogenetic movements and changes in cell fate that transform an asymmetric cluster of cells into an assembly of demarcated and distinct cell types. Gastrulation movements include complicated and well-arranged interactions that generate three germ layers and then contribute to organ formation. However, the study of gastrulation and axial extension is difficult to accomplish *in vivo* due to significant mechanical and geometrical inputs. Gastruloids are helpful experimental systems for studying early developmental patterning mechanisms regulating the acquisition of cell fates, the interrelation of morphogenesis and cell fate determination (Simunovic and Brivanlou, 2017).

Gastruloids are multicellular models of a gastrulating embryo. van den Brink et al. showed that a defined number (approximately 300 cells) of mESCs aggregated to self-organize into polarized structures (a median of 30% of aggregates) that mimic the morphogenetic events of early mouse embryos, including symmetry breaking, the polarization of gene expression, primary axis formation, elongation, and associated patterning. These behaviors *in vitro* are signal-specific and uncouple processes that are tightly associated in the embryo (van den Brink et al., 2014). In other words, mESCs can generate gastruloids in the absence of extraembryonic tissues and almost all relevant signaling cues. Gastruloids display well-organized, distinct gene expression domains demarcating the emergence of the three body axes, anteroposterior axial elongation, and implementation of collinear *Hox* transcriptional patterns over 5–7 days of culture (Baillie-Johnson et al., 2015; Turner et al., 2017; Beccari et al., 2018).

The initial localization of the primitive streak (PS) and its specification after a sequence of events require the specification and localization of the anterior visceral endoderm (AVE) to the future anterior region of the conceptus. By comparing the differences and similarities between gastrulation embryos and gastruloids, researchers have found that gastruloids showed a stable axis which could be initiated without external influences. Thus, these researcher have hypothesized that the true role of AVE was not to break the symmetry of the embryo but rather to ensure that an event that can happen spontaneously is repeatable (van den Brink et al., 2014).

In contrast, measuring the gastruloid formation efficiency (GFE) through a high-performing assay can discriminate different states of pluripotency. The GFE decreases as the pluripotency progresses from a naïve to a primed state. Primed pluripotent cells, such as EpiSCs, fail to generate proper cell aggregates, while early-prime EpiSC-like cells (EpiLCs) aggregate but finally maintain spheroidal morphology (Cermola et al., 2021).

Although much of the signaling network underlying anterior-posterior symmetry breaking has been elucidated in mice, there is little knowledge of axis formation in humans. Etoc et al. generated human gastruloids using colonies of hESCs that grew on micropatterned substrate that geometrically confines the size of cell colonies to generate reproducible fate ordering and differentiation with BMP4 (Etoc et al., 2016). The gastruloids recapitulate the embryonic arrangement of germ layers. The pathways and small molecules functioning in gastruloids provide cues to understand the mechanisms of gastrulation (Etoc et al., 2016). Another research group used hESCs to generate an *in vitro* 3D model of human epiblasts that was similar to E10 epiblasts in terms of size, cell polarity, and gene expression (Simunovic et al., 2019). These researchers showed that symmetry breaks spontaneously despite no prominent asymmetry in ligand application. A defined dose of BMP4 spontaneously induced symmetry breaking and the expression of PS and epithelial-to-mesenchymal transition (EMT) markers.

Gastruloids exhibit similar aspects of early development and body plan formation and provide a more comprehensive knowledge of gastrulation. Gastruloids might also provide an additional test of the developmental fidelity of the cells. As earlier studies have shown, gastruloids appeared to uncouple processes that are tightly linked in the natural embryo (van den Brink et al., 2014). However, whether these processes are truly independent and uncoupled needs further study and new tests are needed for that ESCs are free from some limitations of normal development and can select pathways for differentiation. (Brickman and Serup, 2017).

In contrast, the development of quantitative platforms to pinpoint molecular and subcellular events can further improve the technologies of embryoids, organoids and gastruloids, which promotes a more comprehensive understanding of the complex events of human embryo development (Simunovic and Brivanlou, 2017).

## Discussion

In conclusion, this review discusses the research advances in gametogenesis and embryogenesis using PSCs and their potential for clinical application. The generation of stem cell models and organoids including PGCLCs, *in vitro* induced (IVI) gametes and embryoids, brings a new perspective on human germ cell and embryo development. By combining *in vivo* natural structures and animal models with these organoids, we can systematically investigate the basic principles of gametogenesis and embryogenesis. However, there remain many limitations, such as the time-consuming culture of organoids, the significant differences between organoids and natural structures in terms of size, transcriptomes, epigenomes and lineage specification, the differences between embryo models generated by different laboratories, the low formation efficiency, and the difficulty of maintaining these models for a long time, which need further investigation and improvement in the next couple of years. Gaining more knowledge about development and the emergence of new organoid techniques may help resolve these problems. After further research, IVI gametes obtained from hPGCLC differentiation and other methods can provide opportunities for fertility preservation and could allow the development of a model platform for chemical screens and toxicity tests (Easley et al., 2013). However, there are many ethical and safety issues that need further examinations using more animal species, particularly NHP models.

Because organoids have the advantage of accessibility, the capability of manipulating their gene, and the potential control of variables, as well as rapid technology development, organoids have great potential to become a fundamental model to uncover the principles of gametogenesis and embryogenesis and to be used in clinical translational research. The rapid development of organoid systems promotes clinical translational research on human organoids, which will promote the understanding of physiology and pathophysiology and human organoids can be used to test models and hypotheses generated from studies using animal model systems (Kim et al., 2020). However, although organoids mimic some aspects of human gametogenesis and embryogenesis, they do not recapitulate the whole complexity of these processes and structures. In contrast, because organoids are rather complex systems, the addition of components to these systems to achieve improvement is pretty difficult (Kim et al., 2020). Thus, research of organoids should be based on natural conditions and standards *in vivo*. In the field of reproductive and embryo research, the emphasis on ethics cannot be overstated. We ought to obey the ethical principles and guidelines and stick to the primary societal mission of basic biomedical research and its clinical translation such that our final goal is to alleviate and prevent human suffering caused by illness and injury (Lovell-Badge et al., 2021).

## Abbreviations

BMP4, bone morphogenetic protein 4; bFGF, basic fibroblast growth factor; d4c5 mPGCLCs/GK10FR10/αMEMFBS10, Glasgow’s minimal essential medium (GMEM), 10% knockout serum replacement (KSR), and FR10 for the floating aggregate formation, and αMEM and 10% fetal bovine serum (FBS) for the air-liquid interface culture; EGF, epidermal growth factor; GMEM, Glasgow’s minimal essential medium; IVG, *in vitro* growth culture; IVM, *in vitro* maturation; KSR, knockout serum replacement; LIF, leukemia inhibitory factor; rTestes, d4c5 mPGCLCs [d4 mPGCLCs cultured with forskolin (10 μM), rolipram (10 μM), and cyclosporin A (5 μM) (FR10Cs5) for 5 days] were aggregated with E12.5 embryonic testicular somatic cells; rOvarioids, mPGCLCs are aggregated with FOSLCs, and the mPGCLCs generate functional oocytes from the reaggregates; rOvaries, reconstituted ovaries, mPGCLCs (XX) are aggregated with E12.5 female gonadal somatic cells; rOvarioids, mPGCLCs are aggregated with FOSLCs, and the mPGCLCs generate functional oocytes from the reaggregates; SCF, stem cell factor; TGFb, transforming growth factor β; xrOvaries, xenogeneic reconstituted ovaries, coculture of hPGCLCs with mouse embryonic ovarian somatic cells; 2i, inhibitors of the mitogen-activated protein kinase (MAPK) pathway and the glycogen synthase kinase 3 (GSK3) pathway; 4i, inhibitors of four kinases, namely MAPK, GSK3, p38 and c-Jun N-terminal kinase.
